# Lovastatin Decreases the Expression of CD133 and Influences the Differentiation Potential of Human Embryonic Stem Cells

**DOI:** 10.1155/2016/1580701

**Published:** 2016-05-10

**Authors:** Ade Kallas-Kivi, Annika Trei, Toivo Maimets

**Affiliations:** Institute of Molecular and Cell Biology, University of Tartu, Riia 23, 51010 Tartu, Estonia

## Abstract

The lipophilic statin lovastatin decreases cholesterol synthesis and is a safe and effective treatment for the prevention of cardiovascular diseases. Growing evidence points at antitumor potential of lovastatin. Therefore, understanding the molecular mechanism of lovastatin function in different cell types is critical to effective therapy design. In this study, we investigated the effects of lovastatin on the differentiation potential of human embryonic stem (hES) cells (H9 cell line). Multiparameter flow cytometric assay was used to detect changes in the expression of transcription factors characteristic of hES cells. We found that lovastatin treatment delayed NANOG downregulation during ectodermal and endodermal differentiation. Likewise, expression of ectodermal (SOX1 and OTX2) and endodermal (GATA4 and FOXA2) markers was higher in treated cells. Exposure of hES cells to lovastatin led to a minor decrease in the expression of SSEA-3 and a significant reduction in CD133 expression. Treated cells also formed fewer embryoid bodies than control cells. By analyzing hES with and without CD133, we discovered that CD133 expression is required for proper formation of embryoid bodies. In conclusion, lovastatin reduced the heterogeneity of hES cells and impaired their differentiation potential.

## 1. Introduction

Statins have been safely used for lowering cholesterol synthesis thereby preventing atherosclerotic cardiovascular diseases. A growing body of evidence points to the potential effectiveness of statins in ameliorating other medical conditions such as cancer. Statin treatment of cancer patients has been linked to low death rate, longer survival, and lower risk of venous thromboembolism [1, 2]. Several* in vitro* studies exploring the mechanism of statins' function have revealed that in addition to inhibiting the mevalonate pathway, statins affect signalling pathways regulating cell proliferation and apoptosis. Recently, it has been shown that mevalonate pathway inhibition influences epigenetic mechanisms behind oncogenesis [3]. Epigenetic mechanisms have been shown to regulate either directly or indirectly an intense cross-talk between signalling pathways that affect growth, differentiation, and apoptosis. Therefore, the effects of statins could be very wide-ranging, and their impact on various cell types needs thorough investigation.

Human embryonic stem (hES) cells possess multiple unique features, including an unlimited proliferation potential, expression of specific transcription factors, and the ability to differentiate into the three germ cell layers [4–6]. This makes them a valuable tool for studying certain properties of cancer cells. Studies have shown that exposure of hES cells to lovastatin does not affect karyotypically normal hES cells but suppresses growth and induces apoptosis in karyotypically abnormal hES cells and in colorectal and ovarian cancer cells [7, 8]. Such selectivity makes statins attractive candidates for targeting malignant cells during therapy. However, there is a significant gap in our understanding of the mechanism by which statins affect cancerous cells.

Pluripotency of hES cells is maintained by a transcriptional network that is coordinated by the core transcription factors SOX2, OCT4, and NANOG. In addition, pluripotent hES cells express specific glycosphingolipids (GSLs) SSEA-3, SSEA-4, TRA-1-60, and TRA-1-81 on their surface. hES cells maintain expression of these key transcription factors within the narrow limits that permit continuation of the undifferentiated state. During differentiation, the levels of the pluripotency markers gradually decrease, while concentration of differentiation markers goes up. These changes in transcription factor expression are modulated through mechanisms involving epigenetic modifications. Information about the influence of statins on the differentiation ability of hES cells is currently rather limited.

Other less commonly used markers expressed on the surface of hES cells include the transmembrane protein CD133 [9]. Cell-surface CD133 appears to be lost during differentiation of stem cells, although expression of the CD133 proteins and mRNA can be maintained [10]. The functions of CD133 are still poorly defined. It is associated with membrane protrusions and vesicles export [11, 12] and asymmetric division of cells [13, 14]. Of the three described isoforms of this protein [15–18], isoform CD133-2 has been shown to coexpress with *β*-integrin in the basal layer of human neonatal epidermis [17]. Lovastatin has been reported to reduce the levels of the CD133 marker in hematopoietic stem cells and progenitor cells [19]. Lovastatin strongly inhibits cell migration from aortic explants and causes degradation of capillary tubes, suggesting that statins may have antiangiogenic properties [19].

In this study, we investigated whether the lipophilic statin lovastatin can cause alterations in the expression of the transcription factors that regulate pluripotency and differentiation of the hES cells. In addition, we studied the connection between the CD133 cell-surface marker and the differentiation ability of hES cells.

## 2. Materials and Methods

### 2.1. Cell Culture

H9 ES cell line (WA09, National Stem Cell Bank, Madison, WI, USA) was maintained on Matrigel® (BD Biosciences, San Jose, CA, USA) coated plates in a mTeSR*™*1 maintenance medium (STEMCELL Technologies Inc., Vancouver, Canada) according to the manufacturer's specifications. The used medium was changed for new one on a daily basis. After 3-4 days of growth, the colonies were detached mechanically using a micropipette tip. After breaking up the colonies with gentle pipetting, hES cell clumps were plated onto separate new Matrigel-coated plates.

hES cell cultures were expanded for 3-4 days on six-well plates and treated with 20 *μ*M lovastatin or control solution for 48 hours or grown without lovastatin treatment at 37°C in a 5% CO_2_ humidified atmosphere. Normal karyotype of cells was confirmed using G-banding.

### 2.2. Antibodies and Reagents

Anti-NANOG (PE conjugate), anti-OCT4 (Alexa Fluor 647 and PerCp-Cy5.5 conjugate), anti-SSEA-3 (Alexa Fluor 488 conjugate), anti-SOX2 (PerCp-Cy5.5 conjugate), anti-CD184 (PE conjugate), anti-nestin (Alexa Fluor 647 conjugate), and anti-FoxA2 (PE conjugate) antibodies and their isotype control antibodies were purchased from BD Biosciences. Anti-GATA4, anti-OTX2, anti-SOX17, anti-SOX1, anti-HAND1, and anti-brachyury antibodies were purchased from R&D Systems (Abingdon, Oxon, UK). Anti-CD133 (PE conjugate) and anti-PE conjugated magnetic beads were purchased from Miltenyi Biotec GmbH (Bergish Gladbach, Germany). Sodium butyrate, lovastatin, and reagents for differentiation CHIR99021, SB431542, and LDN193189 were purchased from Sigma-Aldrich Chemicals (St. Louis, MO, USA). Lovastatin was activated into active lactone form according to the protocol [20]. The control solution was prepared using the same protocol, but without adding any lovastatin. Only activated lovastatin (5 mM solution in MQ water) was used in the experiments.

### 2.3. Differentiation of hES Cells into Ectoderm, Endoderm, and Mesoderm Lineages and Embryoid Bodies

hES cells with confluence levels of approximately 60–70% (3-4 days after reseeding), both treated with lovastatin and without any treatment, were grown for a further period (48 hours) in separate differentiation medium containing reagents for the initiation of differentiation into ectodermal, mesodermal, or endodermal lineages. For endodermal lineage commitment, the hES cells were treated with sodium butyrate (1 mM in RPMI 1640 medium containing 1xB27, both from Invitrogen, Paisley, UK). After 24 hours, the medium was replaced with new RPMI 1640 (with 1xB27) containing 0.5 mM sodium butyrate and the cells were cultured for a further 24 hours. For differentiation into the mesodermal lineage, 3 *μ*M of CHIR99021 in RPMI 1640 (with 1xB27) medium was added to the cells for 48 hours, with the medium changed for new one every 24 hours. For differentiation into the ectodermal lineage, hES cells were grown in Essential 6*™* medium (Thermo Fisher Scientific) containing 10 *μ*M of SB431542 and 1 *μ*M of LDN193189 (both from Sigma-Aldrich Chemicals) for 48 hours, with the medium changed for new one every 24 hours. Expression of the differentiation markers was assessed on day two.

For the formation of embryoid bodies (EB) a suspension method was used, where hES cells were manually scraped from the Matrigel plates and then 3D embryoid bodies were formed in Essential 6 medium on a low attachment culture plate. The medium was changed for new one after two days. The formation of EB was assessed visually using a microscope with a 37°C heated stage. Formed EB were collected on day five for flow cytometric analysis.

### 2.4. Multivariate Permeabilised-Cell Flow Cytometry Analysis

After harvesting the hES cells using a 0.05% trypsin-EDTA solution (PAA Laboratories, Linz, Austria) and washing them with PBS, single hES cell suspensions were fixed using a 1.6% paraformaldehyde (PFA, Sigma-Aldrich) solution for 10 minutes at room temperature (RT) as we described previously [21]. The cells were washed and stained using a permeabilisation buffer (Foxp3 Staining Buffer Set, e-Biosciences), blocked using a 2% goat serum (PAA Laboratories) in a permeabilisation buffer (15 minutes) and stained with the appropriate antibodies or their isotype control antibodies for 30 minutes at RT. For cell cycle analysis, the cells were stained with DAPI (Sigma-Aldrich Chemicals). Flow cytometry data were acquired with FACSAria using FACSDiva software (BD Biosciences). Cell permeabilisation, fixation and staining, and data acquisition were done on the same day. The populations that were positive or negative for specific markers were selected using density plots according to the population's borders or using specific biological samples (pluripotent or differentiated hES cells) or specific isotype controls.

### 2.5. Separation of CD133+ and CD133− hES Cell Population Using Magnetic Beads Sorting

The hES cells were removed from the Matrigel-coated plates by manual scraping with a micropipette tip in mTeSR1 maintenance medium containing 10 *μ*M of ROCK inhibitor Y-27632 (Tocris Bioscience). For obtaining single cell suspensions, the detached cells were passed through a 30 *μ*M filter and then incubated with anti-CD133 antibodies (PE conjugate) for 10 minutes at 4°C. The cells were then washed with the mTeSR1/ROCK inhibitor medium. The hES cells were then incubated with anti-PE microbeads (Miltenyi Biotec) for 10 minutes at 4°C and washed with mTeSR1/ROCK inhibitor medium, and then the CD133+ cell population was separated using a MS column. Next, the cells were centrifuged; then the medium was changed to Essential 6 medium (Thermo Fisher Scientific) and the cells were allowed to form EB on low attachment plates. Small aliquots of the obtained cell subpopulations were fixed with 1.6% PFA and characterized for the expression of pluripotency markers using flow cytometric analysis.

### 2.6. Statistical Analysis

A one-tailed paired *t*-test with a confidence interval of 95% was performed with GraphPad Prism 4 software. All results are presented as the mean ± the standard deviation.

## 3. Results

### 3.1. Effects of Lovastatin on the Expression of SSEA-3 and the Transcription Factors NANOG, OCT4, and SOX2 in hES Cells

**Figure 1:**
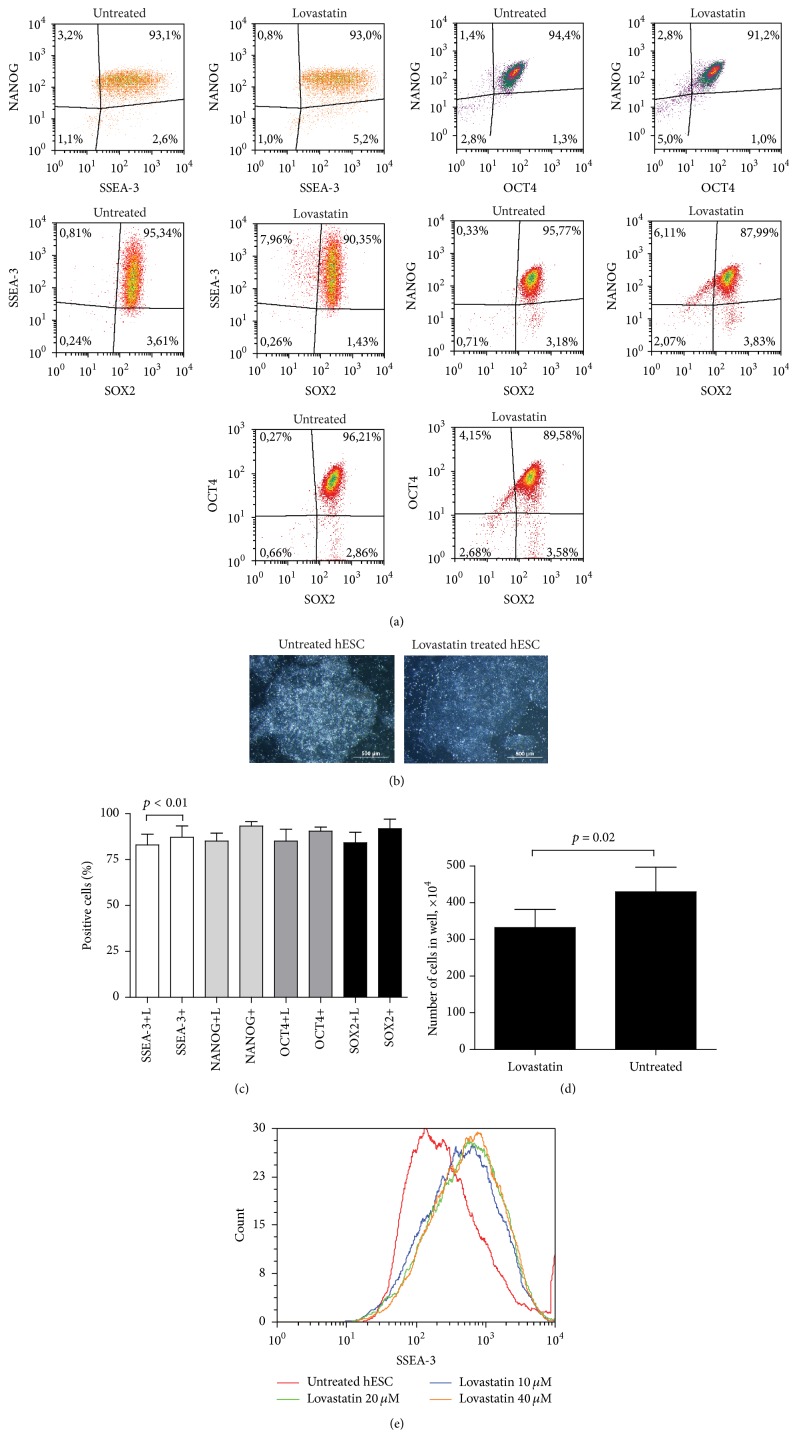
Lovastatin treatment differently affects the expression of pluripotency markers in hES cells. (a) Flow cytometric analysis of the expression of the pluripotency markers NANOG, OCT4, and SOX2 in hES cells treated with lovastatin (20 *μ*M lovastatin for 48 h) and those untreated. Fixed and permeabilised cells were stained with anti-SSEA-3 (Alexa Fluor 488 conjugate), anti-NANOG (PE), anti-OCT4 (Alexa Fluor 647), and anti-SOX2 (PerCp Cy5.5 conjugate) antibodies and with DAPI. For analysis cellular debris and doublets were excluded. (b) The morphological changes in colony structure of lovastatin treated hES cells. (c) The number of cells expressing pluripotency markers. Results are shown as mean ± SD (*n* = 5). (d) The number of cells in one well after 48 h of treatment with lovastatin. (e) Changes in SSEA-3 expression in untreated and lovastatin treated (10–40 *μ*M for 24 h) hES cells.

First, we determined the capacity of lovastatin to modulate pluripotency of hES cells. No significant difference was detected in the expression of the marker SSEA-3 on cell surface or in the levels of the transcription factors NANOG, OCT4, and SOX2 during the first 24 hours of treatment (data not shown). Prolonged exposure (24 to 48 hours) to lovastatin resulted in less compact colony structures, and the total number of cells was significantly lower compared to untreated hES cells (Figures 1(b) and 1(d)). Expression of OCT4, NANOG, and SOX2 decreased (from 90 ± 11% to 84 ± 14%); however, only the drop in the levels of SSEA-3+ (from 90 ± 13% to 83 ± 13%) was statistically significant (Figures 1(a) and 1(c)). SSEA-3 expression in untreated hES cells was highly variable; in hES cells treated with various concentrations of lovastatin this variability decreased along with the increase in lovastatin concentration (10–40 *μ*M), with most cells expressing SSEA-3 at a high level (Figure 1(e)).

### 3.2. Effects of Lovastatin on the Formation of EB

**Figure 2:**
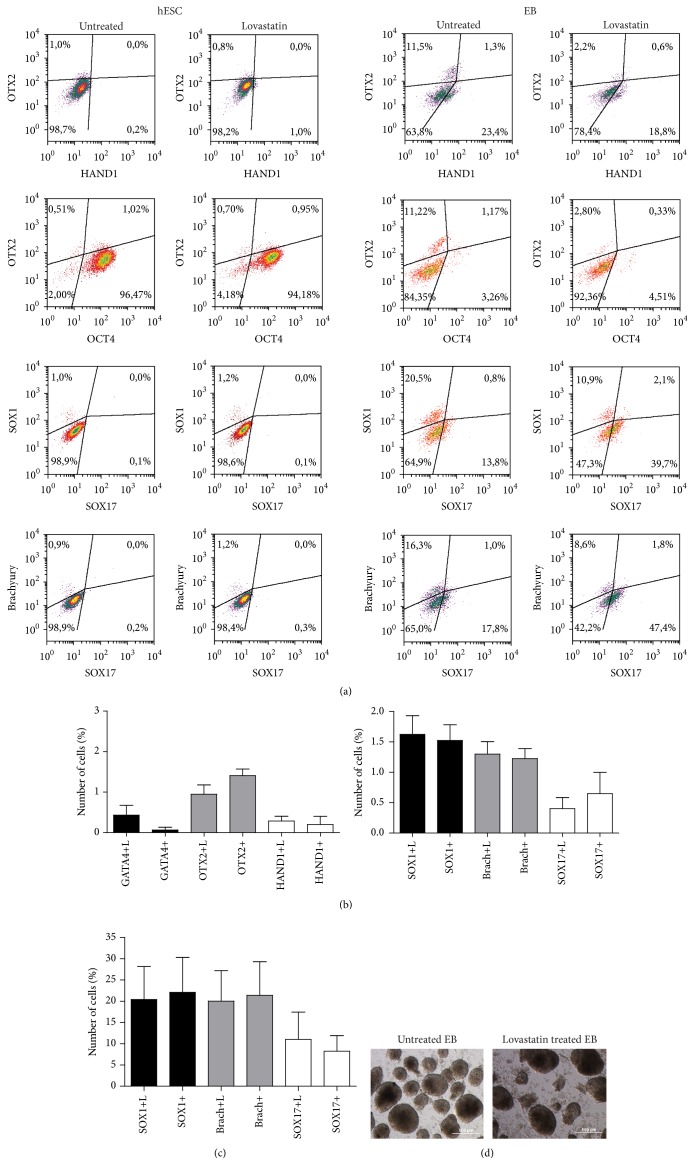
Expression of differentiation markers before and after formation of embryoid bodies from lovastatin treated or untreated cells. (a) Expression of the differentiation markers SOX1, OTX2 (ectodermal lineage), brachyury, HAND1 (mesodermal lineage), and SOX17 (endodermal lineage), and the pluripotency marker OCT4 in hES cells as detected by flow cytometric assay. Embryoid bodies were formed from untreated or 20 *μ*M lovastatin treated hES cells and single cell suspension was used to ascertain the expression of the differentiation markers. (b) Expression of differentiation markers for untreated and lovastatin (L) treated hES cells and (c) of formed embryoid bodies. Results are shown as mean ± SEM (*n* = 4). (d) Morphology of formed EB (on day 5) from untreated and lovastatin treated hES cells.

Next we tested whether the lovastatin treated cells were more likely to form embryoid bodies (EB) under conditions supporting differentiation (e.g., in the absence of bFGF and TGF-*β*). Both treated and untreated hES cells could form EB using the suspension method without any significant changes in their morphology. The number of formed EB was significantly lower when cells were treated with lovastatin (13 ± 3 compared to 20 ± 2 for the controls) (Figure 2(d)). No significant difference in the size of EB between lovastatin treated and untreated cells was detected (136.5 ± 28.9 versus 140.3 ± 31.1). On day five, the EB were collected and single cells were analyzed for expression of the differentiation markers of ectodermal (SOX1 and OTX2), mesodermal (HAND1 and brachyury), and endodermal (GATA4 and SOX17) lineages. Lovastatin treatment had no significant effect on inducing differentiation within the EB (Figures 2(a) and 2(c)). In comparison, we analyzed expression of the same differentiation markers in hES cells before initiation of EB formation. As shown in Figure 2, untreated hES cells expressed high levels of the pluripotency marker OCT4 (90 ± 5% OCT4+ cells), but spontaneous differentiation was very low, with expression of differentiation markers detected in 0.5% to 1.2% of the cells. Similarly low levels of differentiation markers were also detected in lovastatin treated hES cells (Figures 2(a) and 2(b)). Both before and after EB formation, expression of the ectodermal lineage marker SOX1 and mesodermal lineage marker brachyury was detected in cells that were negative for OCT4 (see Suppl. Figure 1 in Supplementary Material available online at http://dx.doi.org/10.1155/2016/1580701). This indicated that expression of this pluripotency factor was downregulated in differentiating cells. Meanwhile, the endodermal marker SOX17 was detected in OCT4 expressing EB cells.

**Figure 3:**
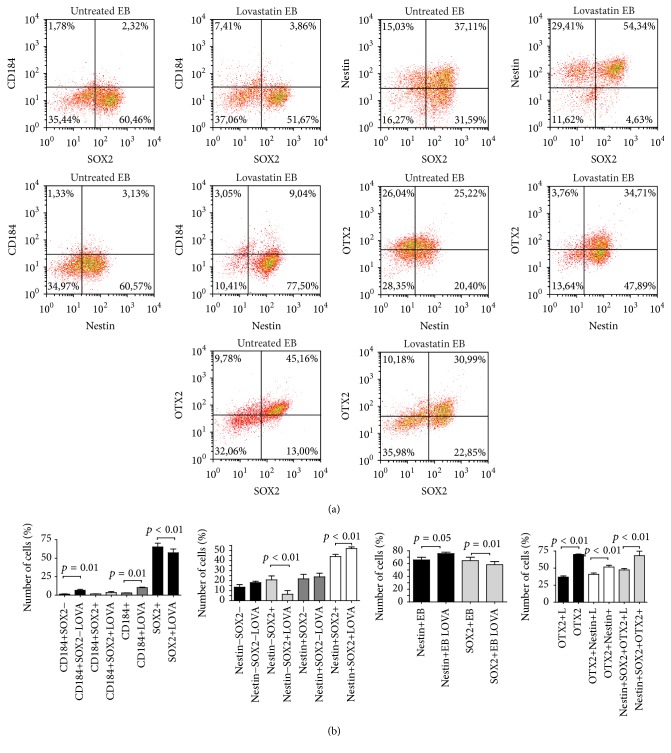
Characterization of untreated and lovastatin treated EB cells for ectodermal differentiation markers. (a) Single cell suspensions from EB were analysed for the expression of CD184 (CXCR4), nestin, SOX2, and OTX2. (b) The mean number of cells ± SD (*n* = 4) expressing the markers.

Since the expression profile of transcription factors that characterize each lineage is time-dependent, we also monitored the levels of CD184, nestin, and SOX2 in the EB cells (Figure 3). Lovastatin treatment resulted in a significant decrease in SOX2 expression (from 65 ± 11% to 57 ± 11%). Expression levels of the differentiation markers CD184 (10 ± 1% versus 3 ± 0.5%) and nestin (75 ± 5% versus 65 ± 9%) were significantly higher in lovastatin treated than in untreated EB cells (Figures 3(a) and 3(b)). A subpopulation of cells was found to express CD184 but not nestin or SOX2. Since CD184 expression has been detected in early endodermal lineage commitment [22], this rare cell population likely represented the cells differentiating to the endodermal lineage. We also noted that the nestin and SOX2 expressing subpopulations were distinct from each other in the cells of EB formed from lovastatin treated hES cells. This could mean that EB formed from lovastatin treated cells were all at a similar stage of differentiation, whereas EB formed from untreated hES cells displayed high heterogeneity in their levels of SOX2 and differentiation markers. For more specific characterization of cells and their neural commitment, we relied on detection of OTX2 in SOX2 and nestin coexpressing cells. Expression of OTX2 was mainly detected in cells that expressed SOX2 and nestin (more than 50% were OTX2+nestin+SOX2+ cells, Figure 3(a)). Lovastatin treatment decreased expression of OTX2 in nestin+SOX2+EB cells (47 ± 4%) compared to EB formed from untreated cells (68 ± 13%).

### 3.3. hES Differentiation into Ectodermal, Mesodermal, and Endodermal Lineages after Treatment with Lovastatin

**Figure 4:**
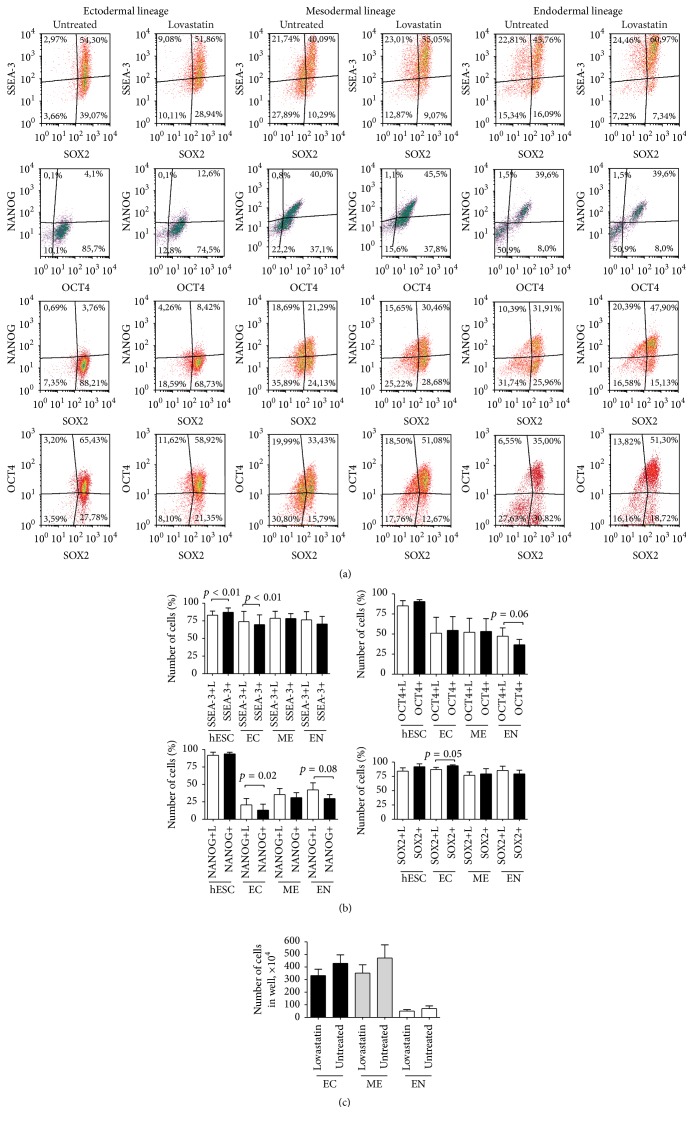
Changes in lovastatin treated and untreated hES cells during differentiation into ectodermal, mesodermal, or endodermal lineages. hEC cells were treated with 20 *μ*M lovastatin for 48 h; then differentiation into ectodermal, mesodermal, or endodermal lineages was initiated by applying special media. After two days of differentiation, the hES cells were collected, fixed, and stained as described in Figure 1. (b) The expression of single pluripotency markers during differentiation into ectodermal (EC), mesodermal (ME), or endodermal (EN) lineages. Results are shown as mean ± SD (*n* = 5). (c) The number of lovastatin treated and untreated hES cells after two days of differentiation (*n* = 5).

In light of the results indicating that the ability of hES cells to form EB was impaired by lovastatin treatment, we used specific differentiation protocols to evaluate any changes in commitment to ectodermal, mesodermal, or endodermal lineages of hES cells treated with lovastatin. First, we analyzed the differences in expression of the pluripotency markers SSEA-3, NANOG, OCT4, and SOX2 during the early stages of differentiation (by day 2). When untreated hES cells were analyzed, the ectodermal and mesodermal induction media resulted in a rapid decrease in NANOG expression (from 93 ± 5% to 19 ± 12%). OCT4 expression remained higher by day two of differentiation (50 ± 12%, Figures 4(a) and 4(b)). During endodermal lineage commitment, expression of both NANOG and OCT4 decreased gradually (from 90% to 31% NANOG+ cells and from 93% to 32% OCT4+ cells, resp.) as shown in our previous report [21]. A distinct regulation of NANOG and OCT4 was detected during the early differentiation events, highlighting the different regulatory role of these transcription factors depending on lineage commitment. In comparison, SOX2 expression remained at a high level by day two (92 ± 6%, Figure 4).

During differentiation into all three lineages, lovastatin treatment inhibited proliferation, with a significantly smaller number of cells detected compared to untreated cultures (Figure 4(c); Suppl. Figure 2). The most significant changes were observed during differentiation into ectodermal and endodermal lineages. During commitment to the ectodermal lineage, lovastatin treated cells displayed significantly higher expression levels of SSEA-3 and NANOG (Figures 4(a) and 4(b)) and significantly reduced expression of SOX2. NANOG expression was more rapidly downregulated in untreated cells differentiating into the ectodermal lineage than in those treated with lovastatin. During commitment to the endodermal lineage, expression levels of NANOG and OCT4 were significantly higher in lovastatin treated cells (45% versus 31% NANOG+ cells and 42% versus 32% OCT4+ cells).

**Figure 5:**
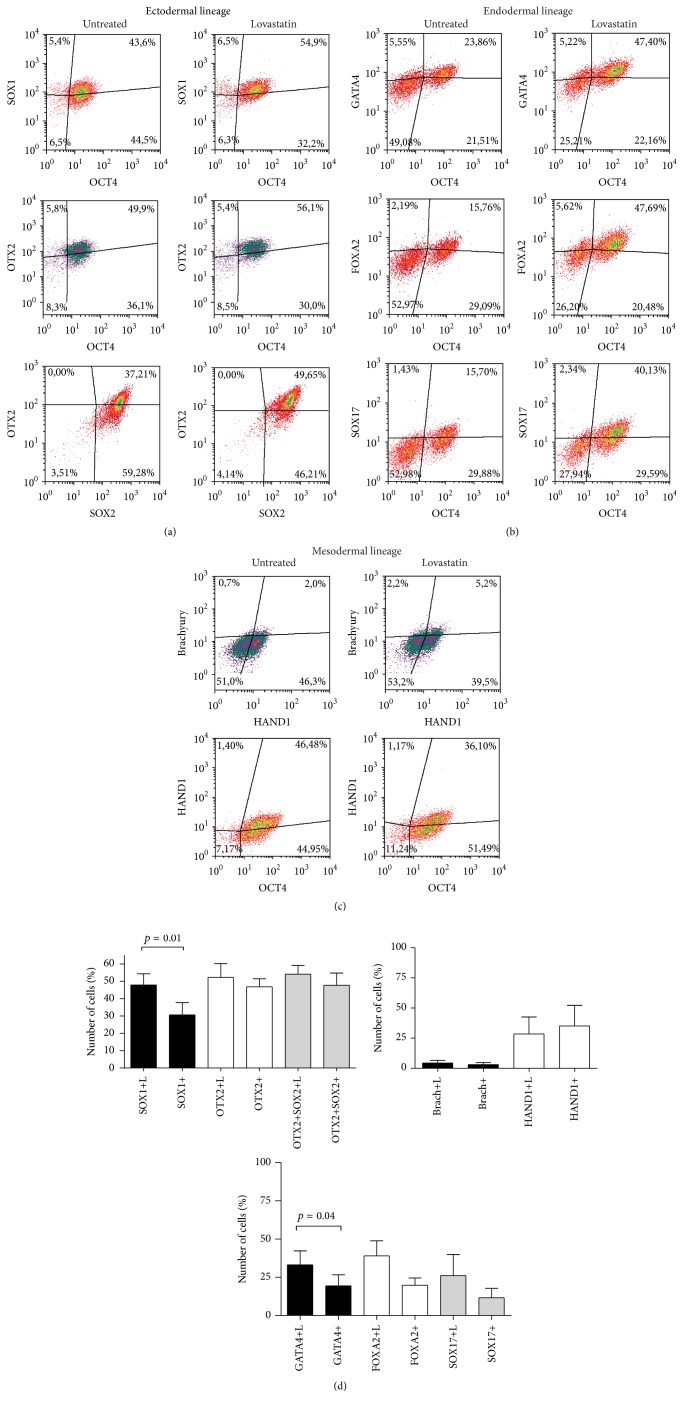
Expression of differentiation markers in hES cells during differentiation into ectodermal, mesodermal, or endodermal lineages after treatment with lovastatin. (a) Expression of the ectodermal lineage markers SOX1 and OTX2 in comparison to the expression of OCT4 and SOX2. (b) Expression of the endodermal lineage markers GATA4, FOXA2, and SOX17 in comparison to OCT4 expression. (c) Expression of the mesodermal lineage markers brachyury and HAND1 and in comparison to the expression of OCT4. (d) The mean number of cells ± SD (*n* = 5) expressing ectodermal (EC), mesodermal (ME), or endodermal (EN) lineage markers.

When analyzing specific differentiation marker expression on day two of differentiation, significant changes resulting from lovastatin treatment were detected in cells differentiating into ectodermal and endodermal lineages (Figures 5(a) and 5(b)). Exposure to lovastatin induced a significantly higher expression of SOX1 (47 ± 12% versus 30 ± 14%) and OTX2 (52 ± 15% versus 46 ± 9%) than untreated hES cells during ectodermal lineage commitment (Figures 5(a) and 5(d)). The number of cells was lower in the lovastatin treated cultures. These cells also expressed higher levels of the pluripotency markers SSEA-3 and NANOG and of the differentiation markers SOX1 and OTX2, suggesting a more efficient differentiation than that taking place in untreated cells. Similarly to the ectodermal lineage differentiation, endodermal lineage commitment was more effective in lovastatin treated cells, with a higher number of GATA4 (33% compared to 19% of GATA4 + cells) and FOXA2 (39% compared to 19% FOXA2+ cells) expressing cells. No significant differences were observed in differentiation marker expression levels in mesodermal lineage differentiating cells (Figures 5(c) and 5(d)).

### 3.4. Lovastatin Decreased CD133 Expression in hES Cells

**Figure 6:**
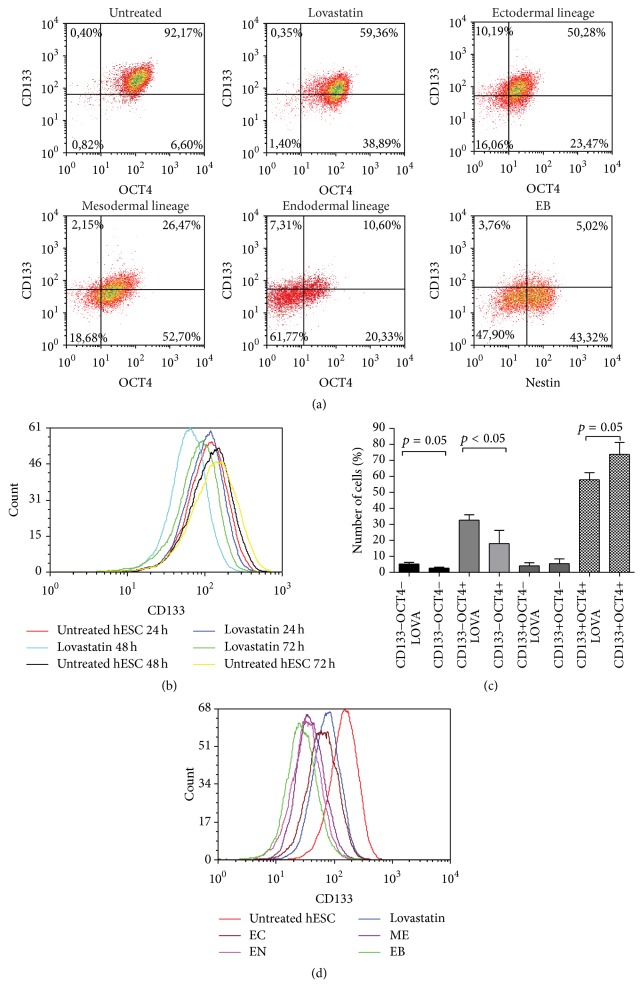
Lovastatin decreased the expression of CD133 in hES cells. Expression of CD133 (AC133 epitope) and the pluripotency marker OCT4 in lovastatin treated hES cells, during differentiation into ectodermal (EC), mesodermal (ME), or endodermal (EN) lineages and after the formation of embryoid bodies (EB). (a) hES cells were stained with anti-CD133 (PE), anti-OCT4 (Alexa Fluor 647), or anti-nestin (Alexa Fluor 647 conjugate) antibodies and with DAPI. For analysis cellular debris and doublets were excluded. (b) CD133 expression levels in untreated and lovastatin (20 *μ*M) treated hES cells at various time points over 24–72 h. (c) Number of cells in each subpopulation according to the coexpression of CD133 and OCT4. Results are shown as mean ± SD (*n* = 5). (d) Histograms of CD133 expression in pluripotent hES cells and differentiating hES cells (untreated and lovastatin treated cells).

Having characterized lovastatin effect on pluripotency and differentiation potential of hES cells, we next investigated its effect on expression of CD133 using the AC133-specific antibody. A 48-hour treatment period with lovastatin (20 *μ*M) decreased CD133 levels in hES cells (from 92% to 59%, Figure 6(a)). In order to clarify whether there was a significant correlation between CD133 and expression of the transcription factor OCT4, the hES cells were fixed and costained with anti-CD133 and anti-OCT4 antibodies. Lovastatin treatment resulted in a significant decrease in the CD133+ OCT4+ cell populations (from 73 ± 17% to 57 ± 10%) and a significant increase in the CD133−OCT4+ cell population (from 18 ± 12% to 32 ± 8%, Figures 6(a) and 6(c)). A longer culturing period (72 hours) revealed that CD133 expression on cell surface increased over time, as did the size of the CD133+OCT4+ cell subpopulation of untreated hES (from 83% to 86%, Figure 6(b)). Lovastatin treatment over the same time period led to a reduced number of cells expressing both CD133 and OCT4 (from 71% to 45%).

To further corroborate the correlation between CD133 and OCT4 levels, CD133 expression was evaluated in hES cells that were differentiated into ectodermal, mesodermal, or endodermal lineages. CD133 levels declined gradually (from 71 to 50%), as did expression of OCT4 (from 90% to 49%), during ectodermal and mesodermal differentiation, confirming the sensitivity of our test system (Figures 6(a) and 6(d)). Differentiation into the endodermal lineage decreased CD133 expression rapidly, with only 10% of cells remaining CD133+. In addition, during differentiation of untreated hES cells into EB, expression of CD133 decreased remarkably (Figure 6(d)). The finding that CD133 levels dropped more rapidly during differentiation into the endodermal compared to other lineages suggested that regulation of this protein's expression is lineage dependent.

### 3.5. Formation of Embryoid Bodies by CD133+ and CD133− hES Cells

In order to clarify the functional differences between CD133 expressing and nonexpressing hES cells, we carried out magnetic bead sorting, which at present is the most harmless cell separation method and therefore the most suitable for the extremely sensitive hES cells. Before separation, we could detect both CD133 expressing (47–52%) and nonexpressing (38–50%) cells (Suppl. Figure 3). The cell populations obtained from magnetic bead sorting using a monoclonal CD133-specific antibody were enriched for CD133+ (up 63%) and CD133− (33%) cells. Despite the presence of the ROCK inhibitor Y-27632, the survival rate of cells in the throughflow and column eluates ranged from 60% to 70%. The process particularly affected cells without CD133 expression, as among this population we detected hES cells without OCT4, SSEA-3, and SOX2 expression (Suppl. Figure 3). In order to characterize CD133 expression relative to OCT4 levels in these subpopulations, we calculated the ratio of the number of CD133+ OCT4+ cells to CD133− OCT4− cells. For the CD133+ cell population this ratio was 7 : 1 and for the CD133− population 1.2 : 1. (In comparison, this ratio was 34 : 1 in lovastatin treated cells, 90 : 1 in untreated cells, and 4 : 1 in hES cells before magnetic bead separation.)

**Figure 7:**
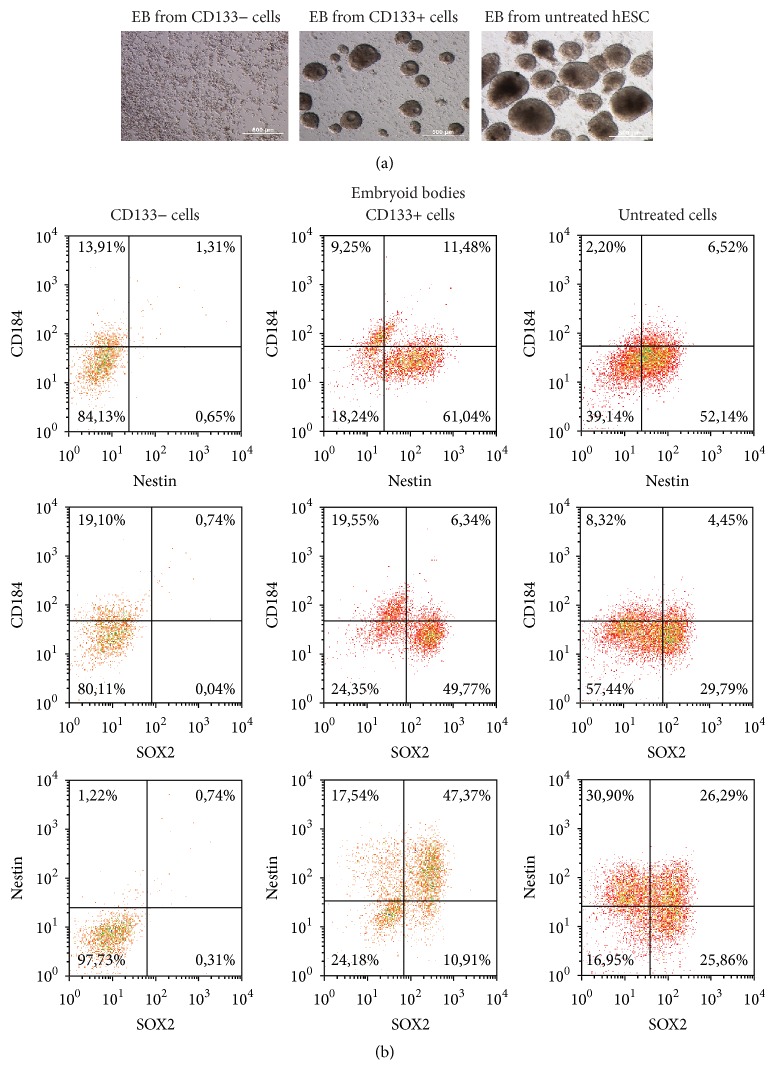
CD133 expression was crucial for proper differentiation into embryoid bodies. CD133+ and CD133− cell subpopulations were enriched using the magnetic beads separation method from untreated hES cells and the formation of EB was initiated. (a) Morphology of the formed structures on day five. (b) Formed EB and cell structures were analysed for the expression of CD184, nestin, and SOX2.

Immediately after magnetic bead sorting, the cells were induced to form EB. Only CD133+ cells were able to give rise to EB. The CD133− cell population formed small aggregates or cell clusters, but no EB were detected (Figure 7(a)). The experiment was repeated twice with the same results. We also induced formation of EB from hES cells before magnetic bead sorting and found that these were larger in size than those from the CD133+ cell population (Figure 7). SOX2 and nestin were detected in CD133+ EB but not in CD133− clusters (Figure 7(b)), highlighting the ineffective differentiation and proliferation of CD133− cells. The fact that only CD133+ hES cells were able to form embryoid bodies demonstrates the crucial role of this protein in the differentiation process.

## 4. Discussion

The growing interest in clarifying statins' mechanisms of action upon various cell types was our reason for analysing human embryonic stem (hES) cells, whose unique properties include differentiating into three germ cell layers. In the current study we found that lovastatin affected hES cells by (i) reducing their expression of CD133; (ii) altering their heterogeneity; (iii) modulating their differentiation potential. Reduced heterogeneity was characterised by lower variability in SSEA-3 expression caused by lovastatin. Furthermore, lovastatin treated hES cells formed EB containing homogenous subpopulations according to nestin and SOX2 expression, whereas untreated hES cells had more heterogeneous subpopulations. The heterogeneity of hES cells is well documented and depends on cell-cell contacts and the microenvironment [23–26]. Differences in the heterogeneity of hES cells could be related to asymmetric division regulated by CD133 [13], whose expression was reduced by lovastatin. Asymmetric division of cells is especially important for stem cells [14] as cells divide asymmetrically during the early stages of division into neural stem cells and neuroepithelial cells, during the latter stages of commitment; however, cells divide symmetrically [27]. Our data describing reduced heterogeneity owing to lovastatin is novel and could be applied to differentiation protocols in order to obtain a more advantageous outcome.

In this study we used lovastatin at concentration of 20 *μ*M that is much higher than dosage for treatment of hypercholesterolemia (plasma concentration in patients ranges from 50 to 250 nM) [28]. It is important to note that lovastatin concentrations for cell culture experiments depend on the type of cell and the specific media, including its components. Different sensitivity to lovastatin has been reported for human vascular smooth muscle cells and microvascular endothelial cells [29]. hES cell growth is supported by special feeder (Matrigel) and its components may have contributed to a nonspecific binding of lovastatin. In patients, lovastatin has a bioavailability less than 5% [28]. Moreover, the effective concentration of lovastatin within cells depends on the expression and regulation of membrane transporters. The efflux ATP-binding cassette transporters ABCB1 (also known as P-glycoprotein) and ABCG2 have an important role in the pharmacokinetics, safety, and lipid-lowering efficacy of statins [16, 17]. Expression of ABCG2 can be detected in several solid and haematological cancer cells and its expression is related to multidrug resistance [23]. In undifferentiated hES cells (HuES cell line), expression of ABCG2 has been reported to be high [30] and needed for the protection of hES cells against xenobiotics and endobiotics. High expression of membrane transporters in hES cells might be one reason why higher concentrations of lovastatin were needed to detect significant changes in the expression of CD133 marker and in the differentiation ability of hES cells.

We used two different approaches for analysing functional ability of treated hES cells to differentiate into the three germ cell layers. Various rearrangements occur in cell structure, morphology, and signalisation networks during the formation of EB [31]. Lovastatin treated cells formed fewer EB; however, we detected the changes only in SSEA-3 and CD133 expression at starting point suggesting that culturing conditions for hES cells before differentiation are crucial. 2D differentiation protocols differ from EB formation, because of using external agents to inhibit particular signalisation pathways. By applying induced differentiation protocols on hES cells we showed enhanced differentiation into ectodermal and endodermal lineages by lovastatin. Additionally we performed very detailed analysis of pluripotency markers NANOG, OCT4, and SOX2 in a commitment into different lineages. A significant reduction in NANOG expression was detected during commitment into the ectodermal and mesodermal lineages, while expression of OCT4 reduced to less than NANOG expression and SOX2 remained high. We concluded that the downregulation of NANOG and OCT4 is lineage dependent during the early stages of differentiation. Gradual downregulation of both NANOG and OCT4 during differentiation into the endodermal lineage was reported in our previous study [21] and confirmed by this study. We showed that SOX2 expression was regulated differently from NANOG and OCT4, since in the ectodermal, endodermal, and mesodermal lineage commitment and in EB cells SOX2 expression remained high. The different levels of transcription factors responsible for pluripotency (OCT4, NANOG, and SOX2) indicated that they likely possess different roles in pluripotent and differentiating hES cells.

We found that CD133 expression correlated positively with OCT4 and SOX2 expression in hES cells. Another research group has shown coexpression of CD133 and OCT4 in hES cells [9]. In the current study, a gradual downregulation of CD133 was detected in OCT4 expressing hES cells during differentiation into the ectodermal lineage. Similar results were obtained during mesodermal induction, indicating that changes in the CD133 epitope recognized by the antibody AC133 occur during an earlier time point of differentiation than changes in the expression levels of OCT4. Since CD133 was detected using an AC133 epitope-specific antibody, then it might be that the particular epitope was not detectable by antibody due to conformation changes as a result of glycosylation. It has reported that loss of the AC133 epitope during the differentiation of hES cells was not correlated with loss of the CD133 protein and mRNA expression [10]. Surface CD133 is decreased by rearrangements in the cell membrane and by the releasing of vesicles containing CD133 as shown in carcinoma cells that express CD133 endogenously at high levels [13]. Whether the decrease in CD133 expression caused by lovastatin was related to the formation of vesicles or due to changes in conformation (glycosylation) of the CD133 epitope during differentiation needs to be addressed further. It might be that different mechanisms are dominant at different time points during differentiation.

By altering CD133 expression, lovastatin may change the epigenetic programme of hES cells. The CD133 intracellular domain has been reported to form a complex with histone deacetylase (HDAC) 6 and regulate the epigenetic programme of cells [32]. It has been reported that in cancer cells the targeting of histone deacetylase 6 (HDAC6) expression or its activity reduces CD133 stability and signalling, resulting in reduced tumorigenesis [32]. We used for endodermal lineage induction sodium butyrate, which inhibits HDACs [33]. CD133 expression also reduced significantly during hES cell endodermal lineage differentiation indicating a link between CD133 downregulation and inhibition of HDACs.

We characterized the different ability of CD133+ and CD133− cells to form EB. The CD133− cell population did not form EB, confirming the importance of CD133 for arranging proper 3D structure and differentiation into the ectodermal lineage. This finding emphasizes the importance of CD133 expression on OCT4 expressing cells in terms of proper differentiation ability. By applying a coculture method, another research group showed that CD133+GFP+ cells followed an ectodermal lineage commitment, while differentiated CD133−GFP+ hES cells predominantly stained positive for endodermal and mesodermal markers [9]. Cancer cells with or without the CD133 expression possess different properties [34, 35]. When we analyzed the CD133− cell subpopulation under conditions for EB formation, SOX2 and nestin were detected in CD133+ EB but not in CD133− cell clusters, highlighting the ineffective differentiation and proliferation of CD133− cells. Since in hES cells SOX2 expression is related to the proliferation potential of cells [21] and nestin is a marker of mitotically active cells [36], then CD133− cells possessed significantly lower proliferation capability. By using magnetic bead separation method, we cannot exclude the possibility that the magnetic beads modulated the differentiation potential of the hES cells. Further analysis to ascertain the most suitable method for hES cell sorting for functional experiments is needed. The remarkable difference in CD133− expressing and nonexpressing cells to form EB found in our study indicates that the ratio of the number of CD133+OCT4+ cells to CD133−OCT4− cells before differentiation is a predictive marker for characterizing the ability of hES cells to form EB.

For ectodermal lineage commitment we used SB431542 and LDN193189, which inhibit ALK4/5/7 [37] and ALK2 and ALK3 [38], respectively. Since lovastatin treatment resulted in higher expression of ectodermal markers, lovastatin could have an additive effect by inhibiting TGF-*β* and BMP signalization pathways. Another research group has shown an inhibitory effect of lovastatin on the TGF-*β* pathway in another cell line [39]. The observation that TGF-*β* is capable of upregulating CD133 expression within the Huh-7 hepatocellular carcinoma cells in a time- and dose-dependent manner suggests a relationship between TGF-*β* signalization and CD133 expression [40]. In addition, CD133 expression is detected on putative cancer stem cells of a variety of solid tumors, being one of the reasons for the chemoresistance of tumor cells [41]. Therefore targeting CD133 using lovastatin offers a promising approach to reduce the heterogeneity of cancer cells and decrease their resistance to chemotherapy. It is important to emphasize that hES cells and cancer cells with stem-cell-like (CSC cells) properties possess multiple differences: (i) CSC cells express several isoforms of OCT4 and splicing variants of NANOG [42, 43], and their role in maintaining stem-cell-like properties remains unclear; (ii) CSC cells have limited differentiation ability. The results of this study are hES specific. In our previous report [44] we compared the response to CDK2 inhibitors in hES and embryonal carcinoma (hEC) cells and showed the different sensitivity of these cell types [44]. The effect of lovastatin on hEC cells and other cancer cell lines with stem-cell-like properties needs further investigation in order to provide evidence for the use of lovastatin to effectively eliminate CSC cells.

## 5. Conclusions

An increasing number of preclinical and clinical studies have shown that statins can affect cancer cells. Therefore, understanding the molecular mechanism of lovastatin function in different cell types is critical to effective therapy design. In this study, we focused on characterizing the effects of lovastatin on pluripotent human embryonic stem (hES) cells. Specifically, we focused on expression of transcription factors characteristic of hES cells and on the ability of these cells to differentiate. We observed that treatment with lovastatin led to impaired differentiation of hES cells into ectodermal and endodermal lineages and to decreased formation of embryoid bodies. We noted that lovastatin caused reduced expression of CD133 on the surface of hES cells. In addition, we demonstrated that high-level expression of CD133 in hES cells was required for proper formation of embryoid bodies. The results of this study provide valuable information regarding the ability of lovastatin to affect stem cells and their differentiation potential.

## Supplementary Material

S Figure 1. Expression of differentiation markers and the pluripotency marker OCT4 in hES cells and formed embryoid bodies. hES cells were treated with 20 μM lovastatin for 48 h, then a sample of the hES cells were analyzed for the expression of differentiation markers and OCT4, and EB were formed from another cell sample.
S Figure 2. Morphological changes in the colony structures of untreated and lovastatin treated hES cells during differentiation into ectodermal, mesodermal or endodermal lineages.
S Figure 3. Characterisation of CD133+ and CD133- hES cell subpopulations obtained using the magnetic beads separation (MACS) method. (A) Expression of CD133, OCT4, SSEA3 and SOX2 in these cell populations are displayed as dot plots or (B) histograms that show the distribution of cells in terms of the various expression levels of specific markers.
