# Supplementary material for: Lovastatin Decreases the Expression of CD133 and Influences the Differentiation Potential of Human Embryonic Stem Cells
Source: Stem Cells Int. 2016 May 10;2016:1580701. doi: 10.1155/2016/1580701 (PMC4877483; doi:10.1155/2016/1580701)
Supplement: Supplementary file 1 — S Figure 1. Expression of differentiation markers and the pluripotency marker OCT4 in hES cells and formed embryoid bodies. hES cells were treated with 20 μM lovastatin for 48 h, then a sample of the hES cells were analyzed for the expression of differentiation markers and OCT4, and EB were formed from another cell sample. S Figure 2. Morphological changes in the colony structures of untreated and lovastatin treated hES cells during differentiation into ectodermal, mesodermal or endodermal lineages. S Figure 3. Characterisation of CD133+ and CD133- hES cell subpopulations obtained using the magnetic beads separation (MACS) method. (A) Expression of CD133, OCT4, SSEA3 and SOX2 in these cell populations are displayed as dot plots or (B) histograms that show the distribution of cells in terms of the various expression levels of specific markers. [file 1580701.f1.pdf]

Suppl.Fig.1

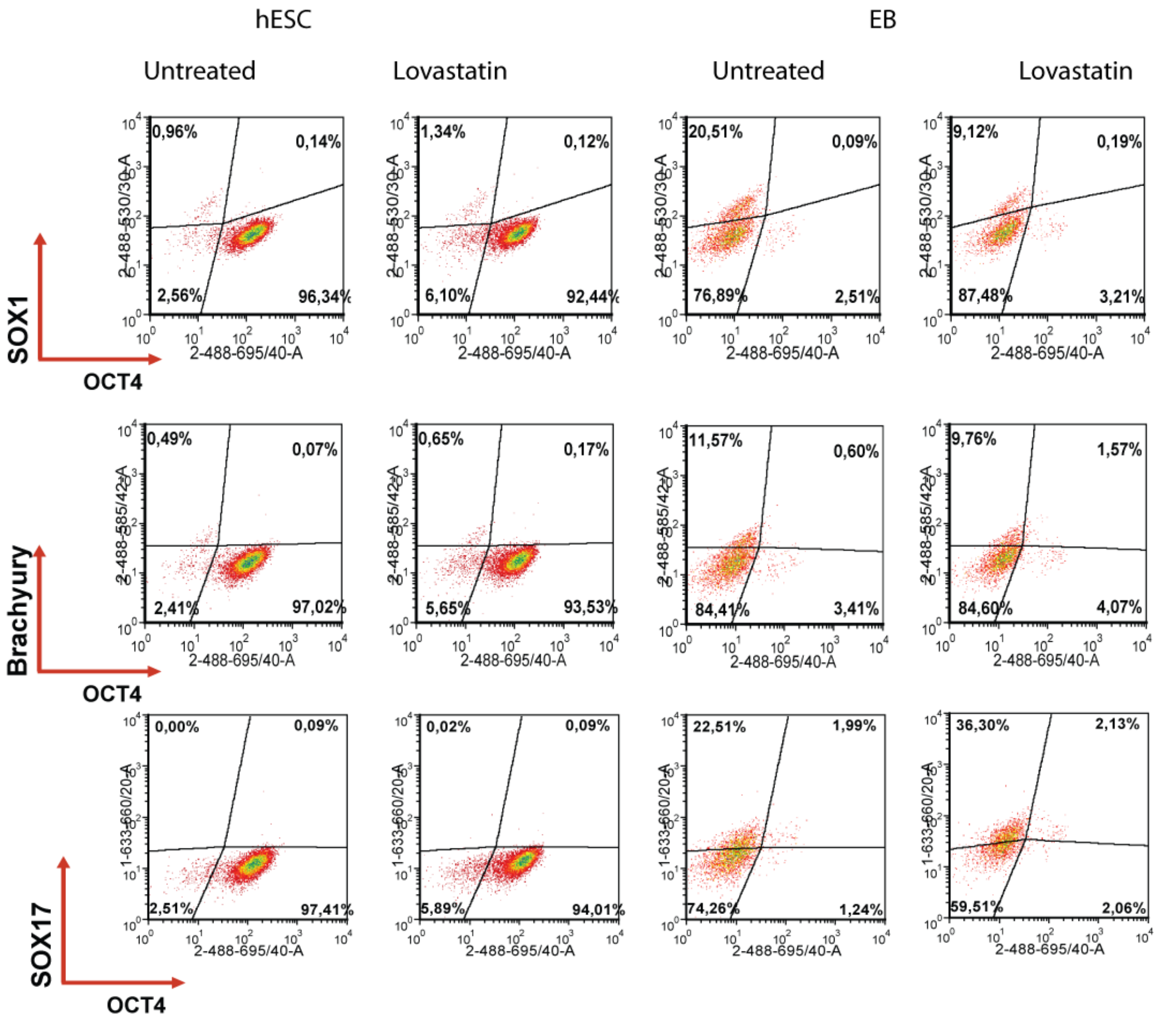
hESC
EB
Untreated
Lovastatin
Untreated
Lovastatin
SOX1
OCT4
Brachyury
OCT4
SOX17
OCT4
0,96%
0,14%
2,56%
96,34%
1,34%
0,12%
6,10%
92,44%
20,51%
0,09%
76,89%
2,51%
9,12%
0,19%
87,48%
3,21%
0,49%
0,07%
2,41%
97,02%
0,65%
0,17%
5,65%
93,53%
11,57%
0,60%
84,41%
3,41%
9,76%
1,57%
84,60%
4,07%
0,00%
0,09%
2,51%
97,41%
0,02%
0,09%
5,89%
94,01%
22,51%
1,99%
74,26%
1,24%
36,30%
2,13%
59,51%
2,06%
2-488-530/30-A
2-488-585/42-A
1-633-660/20-A
2-488-695/40-A

Suppl.Fig.2

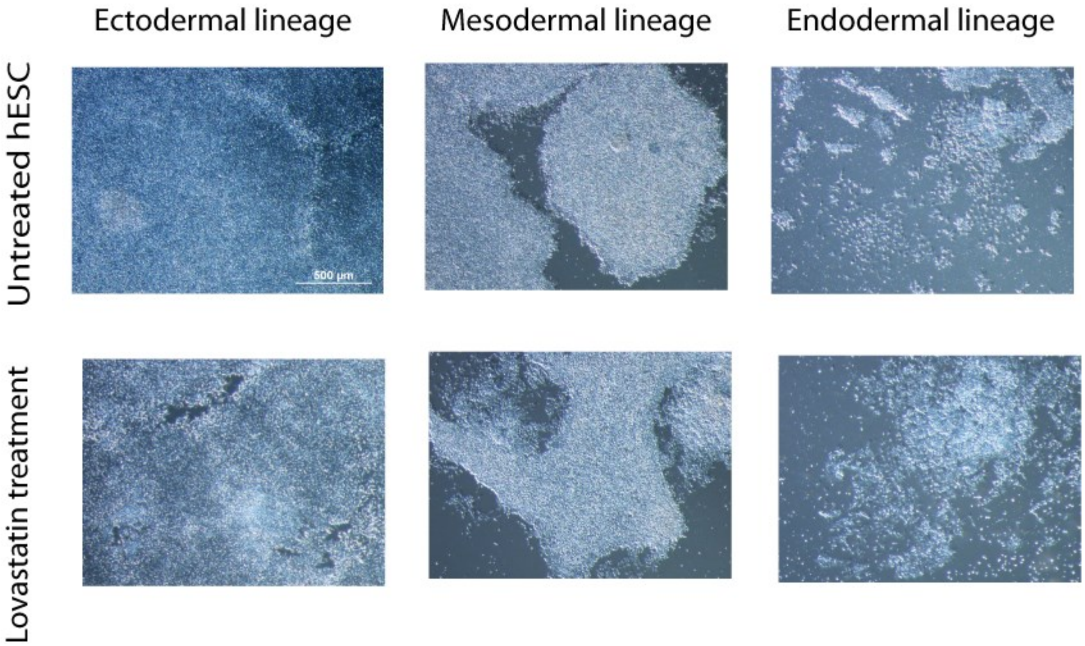
Ectodermal lineage
Mesodermal lineage
Endodermal lineage
Untreated hESC
Lovastatin treatment
500 µm

Suppl.Fig. 3

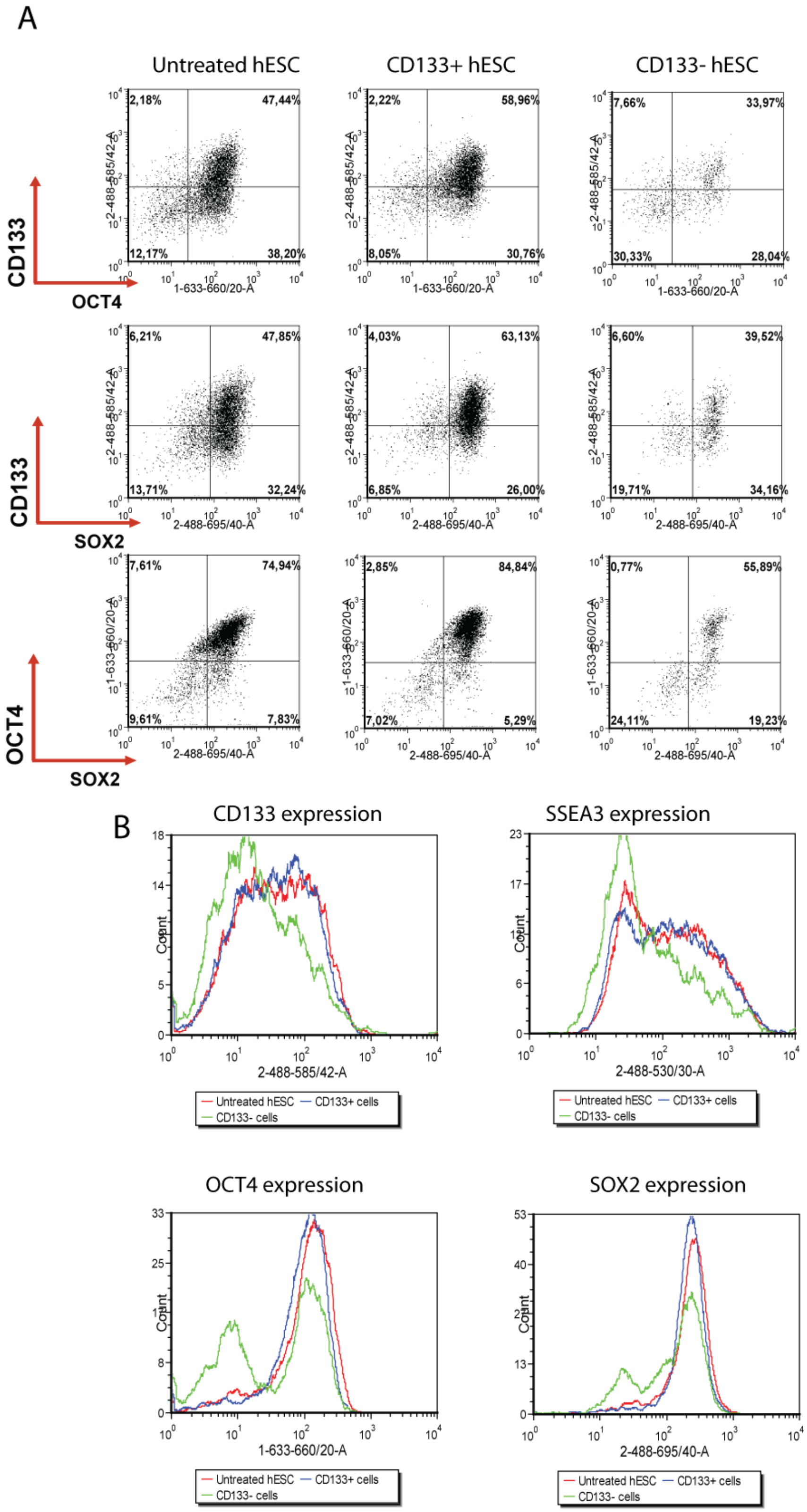
A
Untreated hESC
CD133+ hESC
CD133- hESC
2,18%
47,44%
12,17%
38,20%
2,22%
58,96%
8,05%
30,76%
7,66%
33,97%
30,33%
28,04%
CD133
OCT4
6,21%
47,85%
13,71%
32,24%
4,03%
63,13%
6,85%
26,00%
6,60%
39,52%
19,71%
34,16%
CD133
SOX2
7,61%
74,94%
9,61%
7,83%
2,85%
84,84%
7,02%
5,29%
0,77%
55,89%
24,11%
19,23%
OCT4
SOX2
1-633-660/20-A
2-488-585/42-A
2-488-695/40-A
B
CD133 expression
SSEA3 expression
2-488-585/42-A
2-488-530/30-A
Count
Untreated hESC
CD133+ cells
CD133- cells
OCT4 expression
SOX2 expression
1-633-660/20-A
2-488-695/40-A

Supplementary Materials

Figure 1. Expression of differentiation markers and the pluripotency marker OCT4 in hES cells and formed embryoid bodies. hES cells were treated with 20µM lovastatin for 48h, then a sample of the hES cells were analysed for the expression of differentiation markers and OCT4, and EB were formed from another cell sample.

S Figure 2. Morphological changes in the colony structures of untreated and lovastatin treated hES cells during differentiation into ectodermal, mesodermal or endodermal lineages.

S Figure 3. Characterisation of CD133+ and CD133− hES cell subpopulations obtained using the magnetic beads separation (MACS) method. (A) Expression of CD133, OCT4, SSEA3 and SOX2 in these cell populations are displayed as dot plots (A) or histograms that show the distribution of cells in terms of the various expression levels of specific markers (B).
